# Effect of Polyphenol-Rich Interventions on Gut Microbiota and Inflammatory or Oxidative Stress Markers in Adults Who Are Overweight or Obese: A Systematic Review and Meta-Analysis

**DOI:** 10.3390/nu17152468

**Published:** 2025-07-29

**Authors:** Álvaro González-Gómez, Martina Cantone, Ana María García-Muñoz, Desirée Victoria-Montesinos, Carmen Lucas-Abellán, Ana Serrano-Martínez, Alejandro M. Muñoz-Morillas, Juana M. Morillas-Ruiz

**Affiliations:** 1 Faculty of Pharmacy and Nutrition, UCAM Universidad Católica de Murcia, 30107 Murcia, Spain; agonzalez34@ucam.edu (Á.G.-G.); martinacantone1601@gmail.com (M.C.); clucas@ucam.edu (C.L.-A.); aserrano@ucam.edu (A.S.-M.); jmmorillas@ucam.edu (J.M.M.-R.); 2 Centro de Salud Hellín II, Servicio de Salud de Castilla-La Mancha (SESCAM), 02400 Hellín, Spain; drmunozmorillas@gmail.com

**Keywords:** polyphenols, gut microbiota, obesity, inflammation, oxidative stress, short-chain fatty acids, randomized controlled trials

## Abstract

Background/Objectives: Being overweight and obesity are major public health concerns that demand effective nutritional strategies for weight and body composition management. Beyond excess weight, these conditions are closely linked to chronic inflammation, oxidative stress, and gut dysbiosis, all of which contribute to cardiometabolic risk. Polyphenols—bioactive compounds in plant-based foods—may support improvements in body composition and metabolic health by modulating gut microbiota, reducing oxidative stress, and suppressing inflammation. This systematic review and meta-analysis aimed to evaluate the effects of polyphenol-rich interventions on gut microbiota composition, in combination with either oxidative stress or inflammatory biomarkers, and their potential impact on body composition in overweight or obese adults. Methods: A systematic search of PubMed, Scopus, Cochrane, and Web of Science was conducted through May 2025. Eligible randomized controlled trials included adults (BMI ≥ 25 kg/m^2^) receiving polyphenol-rich interventions, with reported outcomes on gut microbiota and at least one inflammatory or oxidative stress biomarker. Standardized mean differences (SMDs) were pooled using a random-effects model. Results: Thirteen trials (*n* = 670) met inclusion criteria. Polyphenol supplementation significantly reduced circulating lipopolysaccharides (LPSs; SMD = −0.56; 95% CI: −1.10 to −0.02; *p* < 0.04), indicating improved gut barrier function. Effects on cytokines (IL-6, TNF-α) and CRP were inconsistent. Catalase activity improved significantly (SMD = 0.79; 95% CI: 0.30 to 1.28; *p* < 0.001), indicating enhanced antioxidant defense. Gut microbiota analysis revealed increased butyrate (SMD = 0.57; 95% CI: 0.18 to 0.96; *p* < 0.001) and acetate (SMD = 0.42; 95% CI: 0.09 to 0.75; *p* < 0.01), supporting prebiotic effects. However, no significant changes were observed in BMI or body weight. Conclusions: Polyphenol supplementation in overweight or obese adults may reduce metabolic endotoxemia, boost antioxidant activity, and promote SCFAs production. Effects on inflammation and body weight remain unclear. Further long-term trials are needed.

## 1. Introduction

Being overweight and obesity are major public health challenges globally, associated with an increased risk of cardiovascular disease, type 2 diabetes, certain cancers, and all-cause mortality [1,2]. These conditions are closely related to chronic low-grade inflammation and oxidative stress, which contribute to the development of metabolic dysfunction and organ damage [3,4]. A growing body of evidence highlights the key role of the gut microbiota in regulating metabolic health, particularly through its interaction with dietary components and its influence on the host’s immune and oxidative responses [5,6].

Obesity has been associated with a state of gut dysbiosis, characterized by a decrease in microbial diversity and a relative abundance of pro-inflammatory bacterial taxa [7]. This imbalance has been linked to increased intestinal permeability, systemic inflammation, and elevated production of reactive oxygen species (ROS), contributing to oxidative damage to lipids, proteins, and DNA [8]. Therefore, strategies that aim to restore microbial balance and enhance the host’s antioxidant and immunomodulatory defense systems are of significant interest in the prevention and management of obesity-related disorders.

Chronic inflammation in obesity is primarily driven by the expansion of visceral adipose tissue, leading to adipocyte hypertrophy, hypoxia, and the recruitment of pro inflammatory immune cells such as M1 macrophages, contributing to elevated cytokines like tumor necrosis factor-alpha (TNF α), interleukin-6 (IL 6), and C reactive protein (CRP), which are central to insulin resistance, endothelial dysfunction, and hepatic steatosis [3,4,9]. Concomitantly, gut dysbiosis and impaired intestinal barrier integrity may result in metabolic endotoxemia, where translocated lipopolysaccharides (LPSs) engage Toll-like receptor 4 (TLR4), thereby amplifying systemic inflammatory signaling [8,10,11]. This chronic low-grade inflammation significantly contributes to cardiometabolic risk in individuals who are overweight or obese [11].

Polyphenols are a broad class of phytochemicals found in plant-based foods such as fruits, vegetables, tea, coffee, cocoa, and extra virgin olive oil. These compounds have demonstrated antioxidant, anti-inflammatory, and metabolic-regulating properties [12]. Polyphenols and their metabolites can modulate gut microbiota composition by promoting the growth of beneficial bacteria such as *Bifidobacterium* and *Akkermansia muciniphila*, while inhibiting pathogenic species [13,14]. In parallel, polyphenols can reduce oxidative stress by scavenging ROS, upregulating antioxidant enzymes such as superoxide dismutase (SOD), catalase (CAT), and glutathione peroxidase (GPx), and reducing biomarkers of lipid peroxidation, including malondialdehyde (MDA) and F2-isoprostanes [15,16]. Their anti-inflammatory potential involves the inhibition of NF κB and MAPK signaling, as well as the downregulation of COX 2 and iNOS expression [17]. Moreover, polyphenols improve gut barrier function, reduce LPS translocation, and promote the growth of beneficial bacteria, such as *A. muciniphila*, which are inversely associated with inflammation and metabolic risk [8,18,19]. These effects may explain the consistent reductions in circulating inflammatory markers observed in clinical trials using polyphenol-rich interventions [18,20].

Given the modulatory effects of polyphenols on gut microbiota and the established link between microbial dysbiosis, oxidative stress, and chronic inflammation, it has been hypothesized that the health benefits of polyphenols in individuals with obesity may be mediated through concurrent modulation of redox balance and inflammatory processes. In a recent systematic review and meta-analysis, Mao et al. [18] assessed the impact of polyphenols on gut microbiota and inflammatory biomarkers in individuals who are overweight or obese, demonstrating beneficial effects on both outcomes. However, no study to date has systematically reviewed randomized controlled trials that evaluate the impact of polyphenol interventions on gut microbiota composition in combination with either inflammatory or oxidative stress biomarkers. Given the relevance of both pathways in obesity-related metabolic dysfunction, integrating this scattered evidence is crucial.

The aim of this systematic review and meta-analysis is to critically assess randomized controlled trials investigating the effects of polyphenol-rich interventions on gut microbiota, in combination with either oxidative stress or inflammatory biomarkers, in adults who are overweight or obese.

## 2. Materials and Methods

### 2.1. Protocol and Registration

This systematic review and meta-analysis was conducted following the recommendations outlined in the PRISMA 2020 statement [21]. The protocol was prospectively registered in the PROSPERO international prospective register of systematic reviews (CRD420251068835).

### 2.2. Eligibility Criteria

Eligible studies for inclusion in this systematic review and meta-analysis were randomized controlled trials (RCTs), employing either parallel or crossover designs, that investigated the effects of polyphenol-rich dietary interventions on both gut microbiota composition and at least one biomarker of inflammation and/or oxidative stress. Interventions could consist of isolated polyphenolic compounds (e.g., curcumin), complex plant extracts (e.g., grape seed extract), or whole foods with established polyphenol content (e.g., berries, cocoa, sorghum).

Studies were required to include adult participants (≥18 years) with a body mass index (BMI) equal to or greater than 25 kg/m^2^, who are thus classified as overweight or obese. Trials conducted in clinical, community, or free-living settings were considered eligible, provided they included both pre- and post-intervention assessments of gut microbiota composition and at least one relevant biomarker of inflammation (e.g., CRP, IL-6, TNF-α, LPS) or oxidative stress (e.g., MDA, oxLDL, SOD).

Only studies with an appropriate control group, such as placebo, low-polyphenol or phenol-free comparators, or conventional treatment not expected to influence the gut microbiota or inflammation/oxidative status, were included. Exclusion criteria comprised non-randomized studies, trials conducted in healthy-weight individuals or animal models, and studies lacking outcome data on either gut microbiota or inflammatory/oxidative stress markers.

### 2.3. Search Strategy

The systematic literature search was conducted in July 2025 by two independent reviewers (A.M.G.-M. and D.V.-M.) to identify relevant randomized controlled trials examining the effects of polyphenol-rich interventions on gut microbiota and markers of inflammation or oxidative stress in adults who are overweight or obese. The databases searched included PubMed, Scopus, Web of Science, and Cochrane. No restrictions were applied regarding language or publication date to maximize sensitivity and ensure the comprehensive coverage of eligible studies.

The search strategy combined Medical Subject Headings (MeSH) and free-text keywords related to four main domains: (1) polyphenol compounds, including “Curcumin”, “Resveratrol”, “Quercetin”, “Catechins”, “Flavonoids”, and “Phenolic acids”; (2) gut microbiota, including “gut microbiome”, “intestinal flora”, “bacterial diversity”, “Lactobacillus”, and “Bifidobacteria”; (3) inflammation and oxidative stress, including terms such as “C-reactive protein”, “IL-6”, “TNF-α”, “malondialdehyde”, “glutathione”, and “oxidized LDL”; and (4) population and study design, including “Obesity”, “Overweight”, “Body Mass Index”, “Randomized Controlled Trial”, “Clinical Trial”, and “Placebo-Controlled”.

The complete search strategy used for each database is provided in Supplementary Table S1.

### 2.4. Study Selection

Two independent reviewers (D.V.-M and A.M.G.-M) screened titles and abstracts using Mendeley Reference Manager (version 2.109.0). Full texts of potentially eligible studies were retrieved and assessed against the inclusion criteria. Disagreements were resolved by consensus or consultation with a third reviewer. Reasons for exclusion at the full-text screening stage were recorded and are presented in the PRISMA flow diagram.

### 2.5. Data Extraction

Data were independently extracted by two reviewers (D.V.-M. and A.M.G.-M.) using a standardized and piloted data extraction form. The process followed the PICOS framework [22] to ensure consistency and comprehensiveness. Extracted variables included (i) study characteristics (first author, year of publication, country, and study design), (ii) participant characteristics (sample size, mean age, sex distribution, baseline BMI), (iii) intervention details (type of polyphenol, source—food, extract, or isolated compound—daily dosage, and duration), and (iv) characteristics of the comparator or control condition.

Regarding outcomes, the following were recorded: (v) gut microbiota outcomes (e.g., alpha and beta diversity indices, Short-Chain Fatty Acids (SCFAs), relative abundance of specific bacterial taxa, and qualitative compositional changes) and (vi) biomarkers of inflammation (e.g., CRP, IL-6, TNF-α, LPS) and oxidative stress (e.g., MDA, SOD, oxLDL). When multiple time points were reported, only baseline and final post-intervention values were extracted. Any discrepancies in extracted data were resolved by consensus, and, if necessary, by consulting a third reviewer.

### 2.6. Risk of Bias Assessment

The risk of bias of the included randomized controlled trials was independently assessed by two reviewers using the Cochrane Risk of Bias 2.0 (RoB 2.0) tool. This tool evaluates the internal validity of trial results across five domains: (1) bias arising from the randomization process, (2) bias due to deviations from intended interventions, (3) bias due to missing outcome data, (4) bias in measurement of the outcome, and (5) bias in selection of the reported result. Each domain is rated as “low risk”, “some concerns”, or “high risk”, and these judgments are combined to produce an overall risk-of-bias rating for each study. Each study was independently evaluated by two reviewers, with discrepancies resolved by discussion.

### 2.7. Data Synthesis

The primary effect size of the interventions on outcomes related to gut microbiota composition, inflammation, and oxidative stress was quantified using the standardized mean difference (SMD), incorporating Hedges’ g correction to account for small sample sizes. Meta-analyses were conducted using a random-effects model based on the Restricted Maximum Likelihood (REML) method to compute pooled effect estimates with 95% confidence intervals (CIs). When necessary, standard deviations were calculated from reported standard errors, *p*-values, or confidence intervals, following the guidelines provided in the Cochrane Handbook for Systematic Reviews of Interventions [23].

In cases where outcomes were presented only in graphical form, numerical data were extracted using WebPlotDigitizer, version 4.5 [24], with independent verification by two reviewers to ensure accuracy. When multiple time points were reported, only pre- and post-intervention data were used for consistency.

To explore potential sources of heterogeneity, subgroup analyses were performed only for SCFAs, which were measured in serum or fecal samples.

Forest plots were generated to visually present the SMDs and corresponding 95% CIs. Effect sizes were interpreted as small (0–0.20), moderate (>0.20–0.50), or large (>0.50).

Between-study heterogeneity was assessed using the *I*^2^ statistic, with values interpreted as follows: low (<40%), moderate (40–60%), substantial (60–75%), and considerable (>75%) [25]. Potential publication bias and small-study effects were evaluated through funnel plot asymmetry and Egger’s test. A *p*-value of less than 0.05 was considered statistically significant. To assess the robustness of the findings, sensitivity analyses were conducted by excluding studies with a high risk of bias, and the consistency of the results was verified.

All statistical analyses were performed using Stata software (version 19.5; StataCorp, College Station, TX, USA).

## 3. Results

A total of 332 records were identified through systematic searches in four databases: PubMed (*n* = 42), Web of Science (*n* = 128), SCOPUS (*n* = 141), and Cochrane (*n* = 68). After removing 51 duplicates, 328 unique records were screened by title and abstract. Of these, 283 were excluded for not meeting the eligibility criteria.

The remaining 45 full-text articles were assessed for eligibility. After full-text review, 32 studies were excluded for the following reasons: the population was not overweight or obese, BMI/body composition data were not reported [26,27,28,29,30,31,32,33,34,35,36,37], there were insufficient data for meta-analysis [38,39,40,41,42,43], there were no microbiota outcomes [30,44,45,46], there were no control group [47,48,49,50], it was a duplicate publication [51,52,53], there were no oxidative or inflammatory markers [54,55], and the full text was unavailable [25].

**Figure 1:**
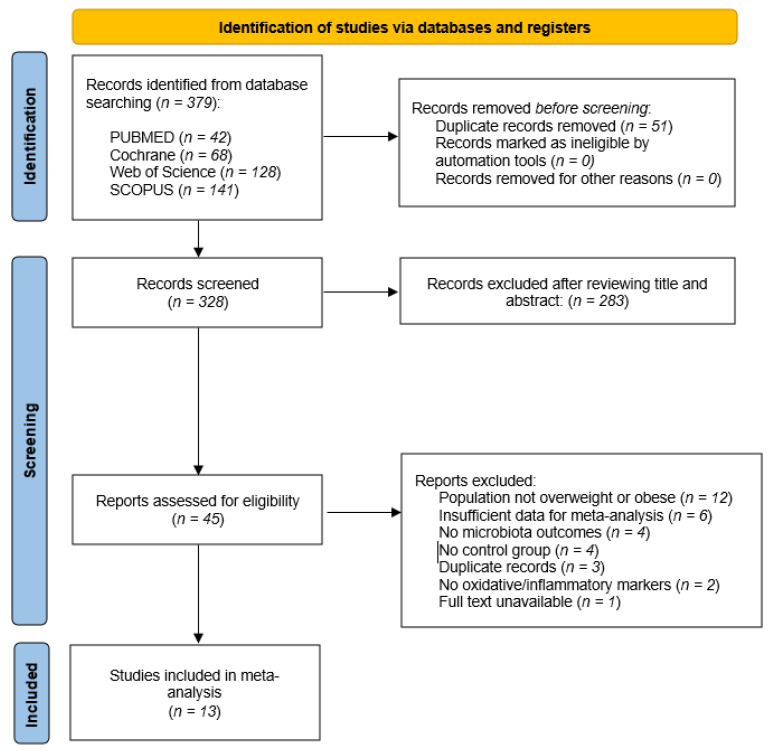
Flow chart.

Ultimately, 13 studies [56,57,58,59,60,61,62,63,64,65,66,67,68] met all inclusion criteria and were included in the meta-analysis (Figure 1).

### 3.1. Study Characteristics

A total of 13 RCTs were included in this systematic review and meta-analysis. These studies were conducted in diverse geographical regions, including Brazil [60,63,64,65], China [59,61], Italy [56,68], the United States [66,67], and one study each from Spain [58], Mexico [57], and South Korea [62]. Sample sizes ranged from 21 to 83 participants, with a combined total of 670 individuals. The duration of the interventions varied from 2 to 24 weeks, with most trials lasting between 4 and 12 weeks.

All participants were adults classified as overweight or obese, with baseline BMI values ranging from 27.3 to 37.4 kg/m^2^, and an overall mean BMI of 30.6 kg/m^2^. The average age across studies was 41.0 years, with ranges between 25.6 and 54.5 years. Sex distribution was generally balanced across studies, although one trial included only male participants [63] and another exclusively female participants [67]. Additionally, one study did not specify this variable [57]. The interventions included a variety of polyphenol-rich sources, such as pomegranate juice [59] or extract [58], genistein [57], silymarin [61,65], yacon flour [64], juçara berry [60], cranberry beverages [66], whole grains [63], *Ecklonia cava* [62], and complex nutraceutical formulations containing fibers, flavonoids, or antioxidant compounds [56,67,68]. Control groups typically received placebo substances such as cellulose or maltodextrin, or non-polyphenolic dietary alternatives (e.g., refined grains or phenol-free beverages).

The primary outcomes assessed included biomarkers of inflammation (e.g., C-reactive protein, interleukins, tumor necrosis factor-alpha, lipopolysaccharide), oxidative stress markers (e.g., MDA, oxLDL, CAT), and gut microbiota-related parameters (e.g., relative abundance of bacterial taxa, short-chain fatty acid concentrations, alpha and beta diversity). Analytical techniques varied across studies and included ELISA, high-performance liquid chromatography (HPLC), gas chromatography–mass spectrometry (GC–MS), 16S rRNA sequencing, quantitative PCR (qPCR), and untargeted metabolomics.

These study characteristics are summarized in Table 1 and Table 2. The substantial variation in intervention types, durations, and outcome assessments highlights the heterogeneity of the current evidence base and supports the value of structured synthesis through meta-analytic techniques.

### 3.2. Effects on Inflammatory Biomarkers

**Figure 2:**
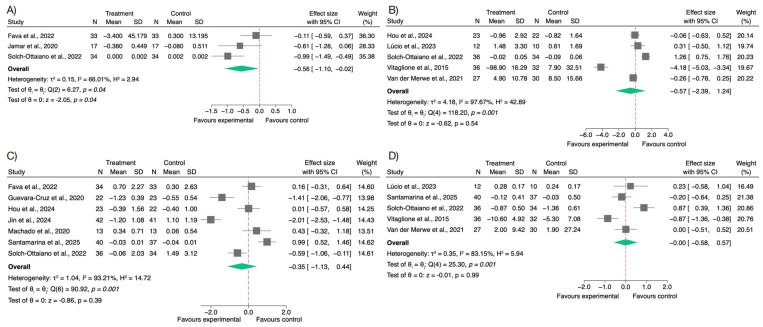
Forest plots of the effects of polyphenol supplementation on inflammatory biomarkers in overweight or obese individuals. (**A**) Lipopolysaccharide (LPS) [56,60,66]; (**B**) Tumor necrosis factor-alpha (TNF-α) [59,63,66,67,68]; (**C**) C-reactive protein (CRP) [56,57,59,61,64,65,66]; (**D**) Interleukin-6 (IL-6) [63,65,66,67,68].

The impact of polyphenol supplementation on systemic inflammation was evaluated across studies by analyzing changes in key pro-inflammatory biomarkers, including IL-6, TNF-α, CRP, and LPS. The meta-analysis revealed a tendency toward anti-inflammatory effects, although the magnitude and statistical significance varied among markers (Figure 2).

A significant reduction was observed for LPS concentrations in participants receiving polyphenols compared to controls (SMD = −0.56; 95% CI: −1.10 to −0.02; *p* < 0.04), indicating improved gut barrier integrity and decreased endotoxemia. This supports a potential gut-mediated anti-inflammatory mechanism of polyphenols. Visual inspection of the funnel plot did not reveal marked asymmetry, suggesting no evidence of publication bias; this was further supported by Egger’s test (β = −1.54; SE = 8.32; z = −0.18; *p* = 0.85). These findings are shown in Supplementary Figure S1.

For CRP, a modest decrease was found (SMD = −0.35; 95% CI: −1.13 to 0.44), though the wide confidence interval reflects high variability and a lack of statistical significance. For this variable, the funnel plot showed some degree of asymmetry; however, this was not confirmed by Egger’s test (β = −0.78; SE = 8.34; z = −0.09; *p* = 0.93), as shown in Figure S1.

Regarding cytokines, the pooled effect on IL-6 was negligible (SMD = −0.00; 95% CI: −0.58 to 0.57; *p* = 0.99), suggesting no consistent modulation by polyphenol supplementation. The funnel plot for this variable did not suggest relevant asymmetry, and Egger’s test confirmed the absence of significant small-study effects (β = 1.63; SE = 5.11; z = 0.32; *p* = 0.75), as shown in Figure S1. The analysis of TNF-α also showed a non-significant decrease (SMD = −0.57; 95% CI: −2.39 to 1.24; *p* = 0.54), with substantial heterogeneity across studies. In contrast, the distribution of studies for TNF-α appeared slightly asymmetric in the funnel plot; however, the result was not statistically significant according to Egger’s test (β = −16.24; SE = 10.72; z = −1.52; *p* = 0.13), as illustrated in Figure S1.

These findings suggest that polyphenol supplementation may contribute to the reduction of certain inflammatory biomarkers—particularly LPS—though the effects on cytokines such as IL-6 and TNF-α remain inconclusive. The high degree of heterogeneity observed (I^2^ ranging from 66% to 98%) warrants cautious interpretation.

Sensitivity analyses excluding studies with a high risk of bias (Santamarina et al. [65] and Van der Merwe et al. [67]) produced results consistent with the primary meta-analysis, thereby reinforcing the validity of the findings (Supplementary Figure S2). No sensitivity analysis was required for LPS, as none of the included studies assessing this marker were classified as high risk of bias.

### 3.3. Effects on Oxidative Stress and Antioxidant Biomarkers

Several studies evaluated the impact of polyphenol supplementation on oxidative stress and endogenous antioxidant defence systems in overweight and obese individuals. The most assessed oxidative biomarkers were MDA and oxLDL, while SOD and CAT were frequently used as indicators of antioxidant enzyme activity [58,61,62,64,66,67].

An increase in MDA levels was observed in the intervention groups (SMD = 0.97; 95% CI: 0.43 to 1.52; *I*^2^ = 13.96%). Since MDA is a stable end-product of lipid peroxidation and a marker of oxidative damage, this unexpected result suggests a potential pro-oxidant effect or may reflect confounding factors such as weight loss-induced lipid mobilization.

For oxLDL, the pooled analysis revealed a reduction trend (SMD = −0.63; 95% CI: −1.60 to 0.34; *p* = 0.20), though the confidence interval included the null value, and heterogeneity was considerable (*I*^2^ = 93.09%). This result, although not statistically significant, aligns with mechanistic evidence suggesting that polyphenols may inhibit LDL oxidation—a key event in the pathogenesis of atherosclerosis and cardiometabolic inflammation.

**Figure 3:**
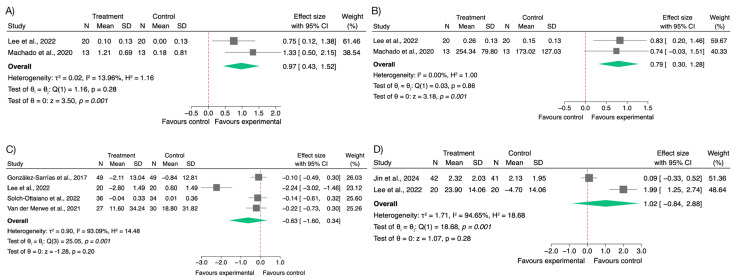
Forest plots of the effects of polyphenol supplementation on oxidative stress biomarkers in overweight or obese individuals. (**A**) Malondialdehyde (MDA) [62,64]; (**B**) Catalase (CAT) [62,64]; (**C**) Oxidized low-density lipoprotein (oxLDL) [58,62,66,67]; (**D**) Superoxide dismutase (SOD) [61,62].

With respect to antioxidant defence, SOD activity showed a notable, though statistically non-significant, increase (SMD = 1.02; 95% CI: −0.84 to 2.88; *I*^2^ = 94.65%; *p* = 0.28), suggesting possible interindividual variability or differences in assay methods. Conversely, CAT activity significantly improved in polyphenol groups (SMD = 0.79; 95% CI: 0.30 to 1.28; *p* < 0.001), with no observed heterogeneity (*I*^2^ = 0%) (Figure 3).

The assessment of publication bias for oxidative stress and antioxidant biomarkers yielded mixed findings. In the case of MDA and CAT, the funnel plot showed no apparent asymmetry, and Egger’s test supported the absence of small-study effects (β = 5.67; SE = 5.26; z = 1.08; *p* = 0.28 for MDA and β = −1.24; SE = 7.28; z = −0.17; *p* = 0.86 for CAT), as depicted in Supplementary Figure S3. In contrast, the distribution of effect sizes for oxLDL and SOD revealed visual asymmetry in their respective funnel plots, which was statistically supported by Egger’s test (*p* < 0.0001 for both; β = −10.88; SE = 2.63; z = −4.14 for oxLDL and β = 11.57; SE = 2.68; z = 4.32), indicating potential small-study effects (Figure S3).

Sensitivity analyses excluding studies with a high risk of bias were conducted specifically for oxLDL, as it was the only oxidative stress marker for which such studies were identified [65,67]. The resulting effect sizes were consistent with those of the primary analysis, indicating that the findings are not substantially influenced by study quality (Supplementary Figure S4).

### 3.4. Effects on Gut Microbiota and Short-Chain Fatty Acids

The modulation of gut microbiota through polyphenol intake was primarily assessed by measuring fecal concentrations of SCFAs, including acetate, propionate, and butyrate. These metabolites are end-products of microbial fermentation and play a critical role in maintaining intestinal barrier function, modulating immune responses, and influencing host metabolism. Variations in SCFAs levels can therefore reflect shifts in microbial composition or activity, indirectly indicating gut health improvements.

Among the three SCFAs analyzed, butyrate showed the most consistent and statistically significant increase following polyphenol supplementation (SMD = 0.57; 95% CI: 0.18 to 0.96; *p* < 0.001).

Acetate levels also increased significantly in the intervention group (SMD = 0.42; 95% CI: 0.09 to 0.75; *p* < 0.01), suggesting a broader microbial stimulation beyond butyrate producers.

In contrast, propionate concentrations remained virtually unchanged between groups (SMD = 0.13; 95% CI: –0.13 to 0.38; *p* = 0.34), suggesting either a limited effect on propionate-producing bacteria or substantial variability in baseline levels and response. The lack of effect on propionate may also be related to differences in polyphenol types or insufficient intervention duration to observe compositional microbial shifts.

**Figure 4:**
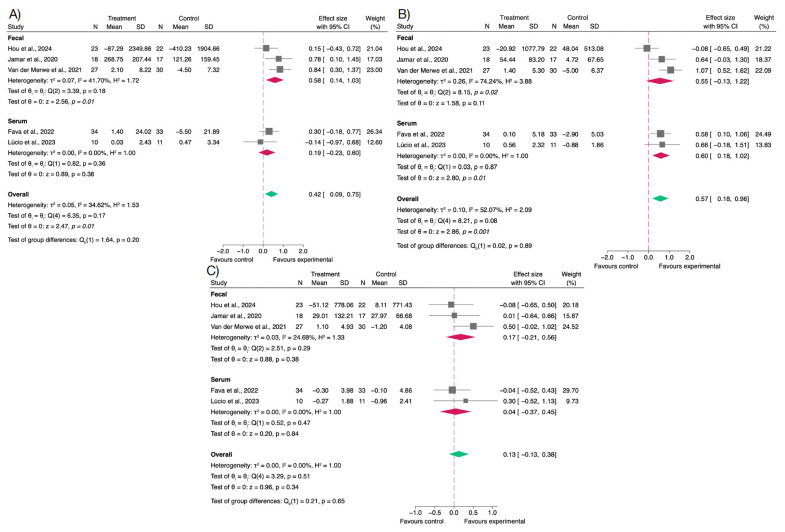
Forest plots of the effects of polyphenol supplementation on short-chain fatty acids (SCFAs) concentrations in overweight or obese individuals. (**A**) Acetate [56,59,60,63,67]; (**B**) Butyrate [56,59,60,63,67]; (**C**) Propionate [56,59,60,63,67].

In summary, these findings indicate that polyphenol supplementation may selectively enhance beneficial microbial metabolites (Figure 4).

Assessment of potential publication bias for SCFAs revealed no major concerns. For acetate and propionate, all studies fell within the expected funnel plot boundaries, and Egger’s test did not indicate small-study effects (β = −2.11; SE = 3.30; z = −0.64; *p* = 0.52 and β = 0.26; SE = 3.76; z = 0.07; *p* = 0.82, respectively). In the case of butyrate, one study lay outside the pseudo 95% confidence limits; however, the funnel plot remained largely symmetrical, and Egger’s test showed no evidence of publication bias (β = 0.59; SE = 2.66; z = 0.22; *p* = 0.95), as shown in Supplementary Figure S5.

The robustness of the results was supported by sensitivity analyses restricted to studies of low or moderate risk of bias (Figure S6 of the Supplementary Material). Overall, effect estimates remained consistent with those of the primary analyses. Notably, heterogeneity in the analysis of butyrate was substantially reduced, indicating greater coherence across studies. For acetate, the exclusion of one high-risk study resulted in a *p*-value above the threshold for statistical significance.

### 3.5. Effects on Body Weight and BMI

Several studies evaluated the effects of polyphenol supplementation on anthropometric parameters, specifically changes in BMI. The pooled analysis of studies reporting weight outcomes showed a small and non-significant effect (SMD = 0.13; 95% CI: −0.43 to 0.69), with high between-study heterogeneity (*I*^2^ = 84.15%). These findings suggest that, overall, polyphenol supplementation may not significantly impact body weight in overweight or obese individuals, although individual studies showed variability in the magnitude and direction of effect.

**Figure 5:**
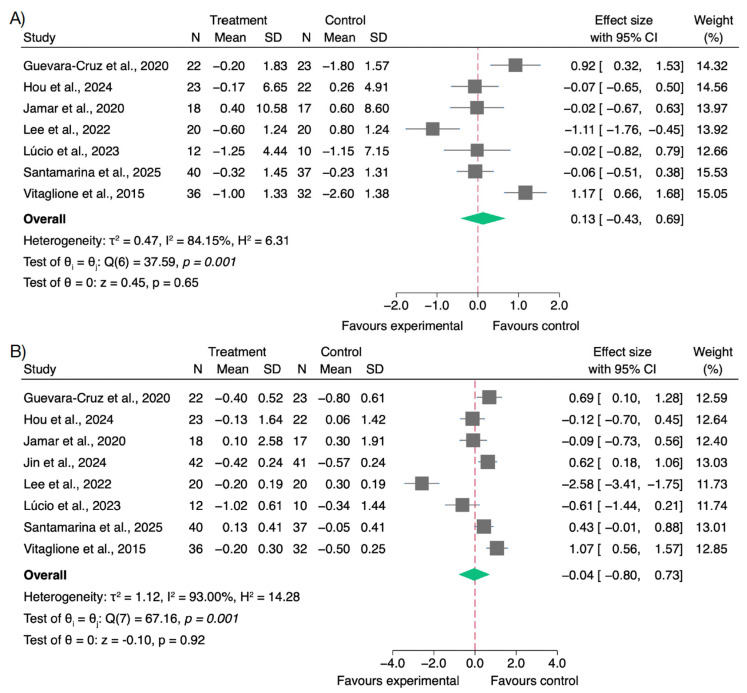
Forest plots of the effects of polyphenol supplementation on body weight and body mass index (BMI) in overweight or obese individuals. (**A**) Body weight [57,59,60,62,63,65,68]; (**B**) Body mass index (BMI) [57,59,60,61,62,63,65,68].

Similarly, for BMI, the meta-analysis yielded a negligible and non-significant pooled effect (SMD = −0.04; 95% CI: −0.80 to 0.73), with substantial heterogeneity across studies (*I*^2^ = 93.00%) (Figure 5). These results indicate that polyphenol interventions, as implemented in the included trials, are unlikely to produce meaningful changes in BMI over short- to medium-term durations.

The wide confidence intervals and heterogeneity observed in both analyses may be due to differences in intervention type, polyphenol dose, baseline BMI, or concurrent dietary and physical activity recommendations.

For body weight, the funnel plot appeared symmetrical, and Egger’s test was not significant (β = −3.83; SE = 5.47; z = −0.70; *p* = 0.48), indicating no signs of publication bias (Supplementary Figure S7). For BMI, slight asymmetry was observed, and Egger’s test suggested potential small-study effects (β = −11.54; SE = 3.30; z = −3.50; *p* < 0.001), as shown in Supplementary Figure S7.

Sensitivity analyses were performed for body weight and BMI by excluding studies with a high risk of bias [65,67]. The pooled estimates remained consistent with those of the primary analyses, confirming that the observed effects were not driven by lower-quality studies (Supplementary Figure S8).

### 3.6. Risk of Bias Assessment

**Figure 6:**
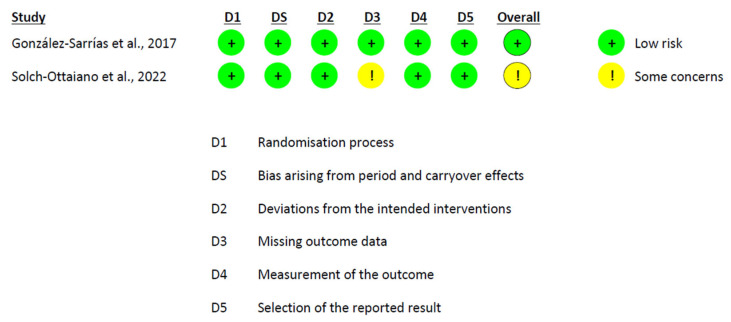
Risk of Bias Assessment in Crossover Trials Using ITT Analysis [58,66].

**Figure 7:**
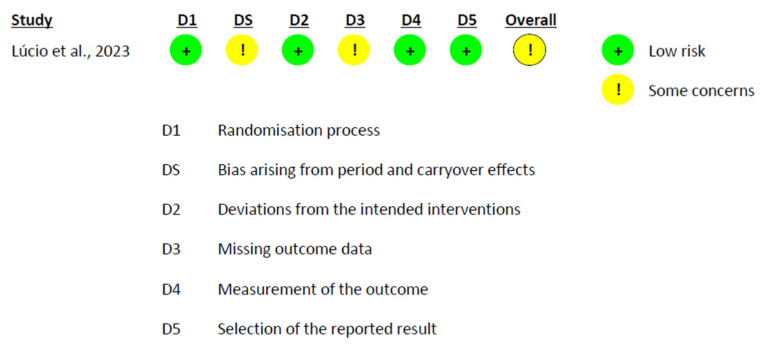
Risk of Bias Assessment in Crossover Trials Using PP Analysis [63].

**Figure 8:**
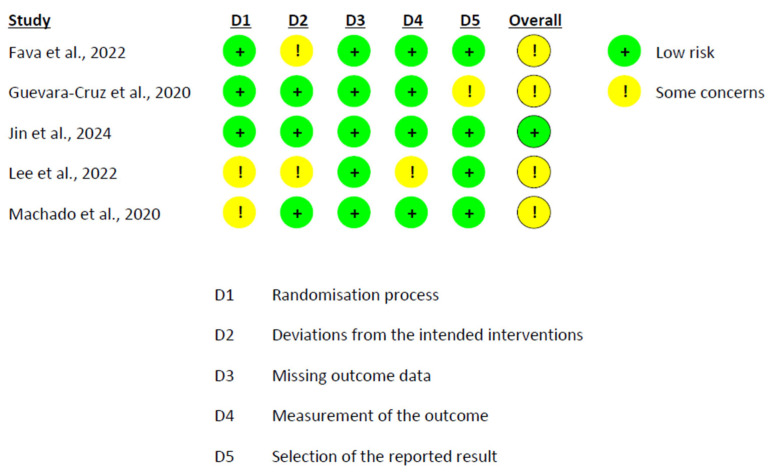
Risk of Bias Assessment in Parallel Trials Using ITT Analysis [56,57,61,62,64].

**Figure 9:**
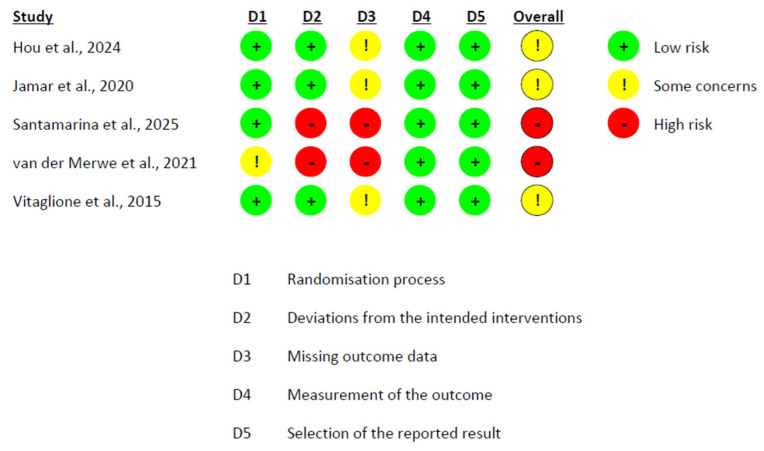
Risk of Bias Assessment in Parallel Trials Using PP Analysis [59,60,65,67,68].

The risk of bias assessment revealed a heterogeneous profile across studies. Among the crossover trials analyzed under the intention-to-treat (ITT) approach, González-Sarrías et al. [58] were rated as low risk across all domains. In contrast, Solch-Ottaiano et al. [66] were categorized as having some concerns, mainly due to missing outcome data that were not clearly addressed (Figure 6). In the per-protocol (PP) evaluation of crossover designs, Lúcio et al. [63] also presented some concerns, particularly due to the potential for carryover effects and unclear handling of missing data (Figure 7). In parallel-group trials analyzed by ITT, Fava et al. [56] showed minor protocol deviations, while Guevara-Cruz et al. [57] raised concerns regarding the selection of the reported results, as the clinical trial registration was not provided. Jin et al. [61] were rated as low risk across all domains. Lee et al. [62] and Machado et al. [64] additionally presented concerns related to the randomization process (Figure 8). In the PP analyses of parallel-group trials, Hou et al. [59], Jamar et al. [60], and Vitaglione et al. [68] were judged to have some concerns, mainly due to issues related to missing outcome data. Conversely, Santamarina et al. [65] and van der Merwe et al. [67] were rated as high risk. In both cases, although randomization and blinding were generally adequate, high attrition and exclusion of participants without applying ITT or imputation methods introduced substantial bias (Figure 9).

## 4. Discussion

This systematic review and meta-analysis assessed the effects of polyphenol supplementation on inflammation, oxidative stress, antioxidant defense, gut microbiota, and anthropometric outcomes in overweight and obese adults. The following sections discuss each outcome in detail.

### 4.1. Modulation of Metabolic Endotoxemia and Inflammatory Status

The observed reduction in circulating LPS found in our meta-analysis (SMD = −0.56; 95% CI: −1.10 to −0.02) is particularly relevant, given that elevated LPS levels are a hallmark of metabolic endotoxemia and reflect impaired gut barrier function and chronic low-grade inflammation. These conditions are pivotal in the pathogenesis of obesity-related metabolic disturbances such as insulin resistance and hepatic steatosis [8,10]. The significant pooled effect suggests that polyphenol-rich interventions may exert a gut-mediated anti-inflammatory effect by improving epithelial integrity and modulating microbial composition.

This interpretation is supported by individual studies included in the review. For instance, Guevara-Cruz et al. [57] reported a marked reduction in serum LPS concentrations after genistein supplementation in obese adults, which was accompanied by increased abundance of *Akkermansia muciniphila* and improved insulin sensitivity through skeletal muscle AMPK activation. Similarly, Hou et al. [59] observed a reduction in LPS levels after pomegranate juice intake, together with favorable shifts in microbial composition (notably *Akkermansia* and *Bifidobacterium*) and SCFAs production, particularly butyrate, which may further enhance intestinal barrier integrity. However, other studies did not replicate this effect. Jamar et al. [60] found no significant reduction in LPS following juçara berry supplementation, despite increases in *A. muciniphila*, *Bifidobacterium* spp., and acetate levels, and Fava et al. [56] reported increases in *Bifidobacterium* and *Lactobacillus* without changes in endotoxemic markers. These discrepancies may stem from differences in polyphenol type, dose, duration, or bioavailability, as well as interindividual metabolic responses.

A recent review by Rębas [69] also supports the role of polyphenols in LPS modulation through epithelial protection and microbial enrichment, while noting the same heterogeneity across studies. Analytical variability (e.g., ELISA vs. LAL assay) may further contribute to inconsistent LPS detection thresholds.

Overall, our meta-analytic evidence reinforces the concept that select polyphenols can mitigate metabolic endotoxemia, likely through a combination of microbial modulation and epithelial reinforcement mechanisms. The observed reduction in LPS supports the therapeutic potential of polyphenol-rich foods and supplements as adjunctive strategies to combat obesity-related inflammation [70], although longer-term studies and standardized protocols are needed to confirm their sustained efficacy and identify the most effective compounds and doses.

Regarding pro-inflammatory cytokines, our pooled estimates showed no significant changes in IL-6 (SMD = −0.12; 95% CI: −0.43 to 0.18) or TNF-α (SMD = −0.57; 95% CI: −2.39 to 1.24). This variability was mirrored in individual trials. Among the included trials, some reported reductions in circulating cytokine levels following polyphenol intake. For instance, Jamar et al. [60] found that supplementation with juçara berry significantly decreased TNF-α levels in obese individuals over six weeks, in parallel with an increase in *Akkermansia* and acetate levels. This suggests that microbial-derived metabolites might mediate part of the anti-inflammatory effect. However, IL-6 levels remained unchanged, highlighting potential selectivity in the inflammatory response. In contrast, Solch-Ottaiano et al. [66] observed no significant changes in IL-6 or TNF-α after a two-week intervention with a high-polyphenol cranberry beverage, despite slight modulation of gut microbiota composition. The authors attributed the lack of effect to the short duration of the trial and the relatively low baseline inflammatory status of the participants, which may have limited the ability to detect improvements. Moreover, interindividual variability in polyphenol metabolism and bioavailability could have contributed to the null findings.

Similarly, the meta-analysis found no significant effect on CRP (SMD = −0.35; 95% CI: −1.13 to 0.44) with substantial variability across studies. Factors such as baseline inflammatory status, intervention length, and polyphenol formulation likely modulate responsiveness, as highlighted in prior meta-analyses [54]. Overall, while select trials indicate anti-inflammatory potential, polyphenol supplementation does not consistently reduce systemic inflammatory markers in overweight or obese adults.

### 4.2. Oxidative Stress Biomarkers and Antioxidant Response

In terms of oxidative stress, the findings of the meta-analysis suggest mixed effects of polyphenol supplementation, depending on the biomarker assessed. Notably, MDA levels increased significantly in the intervention group (SMD = 0.97; 95% CI: 0.43 to 1.52), indicating greater lipid peroxidation and a deterioration of oxidative status rather than the expected improvement. This result contrasts with previous reviews, such as Jomova et al. [71]. The magnitude of the effect observed may reflect the potency of the polyphenols used, their bioavailability, or the baseline oxidative status of the participants. However, it is important to consider that MDA levels may be influenced by multiple factors beyond lipid peroxidation, including elevated glucose concentrations [72], among others. Furthermore, high doses of polyphenols could exert pro-oxidant effects under certain conditions, potentially contributing to paradoxical increases in oxidative stress [73,74]. High doses of polyphenols, while generally known for their antioxidant properties, can indeed exhibit pro-oxidant effects under specific conditions in humans. This pro-oxidant activity is more likely at elevated concentrations, particularly in the presence of transition metal ions or specific physiological environments, and may result in increased oxidative stress markers such as MDA [73,75,76]. Some studies have suggested that excessive polyphenol intake, especially from supplements rather than food-based sources, could interfere with redox homeostasis or even increase oxidative damage, raising important considerations about dose, formulation, and context of use. In addition, certain polyphenols may interfere with glutathione synthesis, reducing endogenous antioxidant capacity and temporarily increasing oxidative damage [77,78].

In contrast, the activity of CAT increased significantly with the interventions (SMD = 0.79; 95% CI: 0.30 to 1.28), supporting a beneficial effect of polyphenols on endogenous antioxidant defence. SOD also showed a favorable trend (SMD = 1.02; 95% CI: −0.84 to 2.88), although with wide variability and a lack of statistical significance. These findings are in line with molecular studies demonstrating that polyphenols activate the Nrf2/ARE signaling pathway, leading to transcriptional upregulation of antioxidant enzymes such as CAT, SOD, and glutathione peroxidase [79,80,81].

The clinical relevance of the observed CAT increase is further supported by trials such as Santamarina et al. [65], who reported enhanced enzymatic antioxidant activity and improved redox balance after polyphenol-based supplementation in overweight individuals. Furthermore, Jin et al. [61] highlighted the modulatory role of gut microbiota in transforming dietary polyphenols into bioactive metabolites with greater antioxidant efficacy, which may partly account for the variability in effects across studies.

Taken together, although the increase in MDA challenges the commonly assumed antioxidant role of polyphenols, it may be interpreted as a temporary rise in oxidative burden followed by adaptive upregulation of protective enzymes—a phenomenon previously observed in hormetic responses [82]. The concurrent enhancement of catalase activity therefore suggests that polyphenol supplementation may contribute to redox homeostasis, not necessarily by lowering oxidative stress markers directly but by enhancing the body’s capacity to neutralize reactive species.

### 4.3. Effects on SCFAs Production

Polyphenols, while not direct substrates for SCFA production, can significantly influence SCFA levels by modulating the gut microbiota. Research has shown that polyphenol supplementation increases the abundance of beneficial bacteria such as *Bifidobacterium*, *Lactobacillus*, *Roseburia*, and *Faecalibacterium*, many of which are known producers of acetate and butyrate, key SCFAs linked to gut and metabolic health [83,84,85,86]. Both animal and in vitro studies have demonstrated that polyphenols derived from sources such as grape seeds, apple peel, and sugarcane can enhance SCFA production, particularly butyrate and propionate, by promoting these microbial populations. The effect appears to be source-specific and may depend on the polyphenol type, the presence of fermentable dietary fibers, and the region of the colon, with some polyphenols favoring acetate production and others influencing butyrate or propionate [87,88,89].

The observed increase in butyrate concentrations was both statistically significant (SMD = 0.57 [0.18, 1.96]) and biologically relevant, given the well-established role in maintaining intestinal epithelial integrity, regulating immune function, and serving as an energy source for colonocytes [90]. These findings reinforce the hypothesis that polyphenols exert prebiotic-like effects, selectively enhancing butyrogenic taxa such as *Faecalibacterium prausnitzii* and *Roseburia* spp. [83,91,92].

Several included trials reported either a direct increase in fecal butyrate levels or an enrichment of butyrate-producing bacteria. For instance, Jin et al. [61] demonstrated that supplementation with a polyphenol-rich plant blend significantly raised fecal butyrate alongside improved gut barrier markers. Similarly, Lúcio et al. [63] observed shifts in microbial composition toward a healthier profile in overweight men consuming extruded sorghum rich in polyphenols, suggesting enhanced fermentative metabolism and SCFAs output.

Acetate concentrations also increased significantly following polyphenol supplementation, although the effect size was more modest compared to butyrate. As the most abundant SCFA, acetate contributes to lipid metabolism, appetite regulation, and intestinal immune balance, and its elevation may reflect a general stimulation of microbial fermentation activity [93,94]. In contrast, propionate levels did not show significant or consistent changes across studies. This variability may stem from its higher absorption rate and greater interindividual response to dietary interventions. The selective impact of polyphenols on microbial metabolism likely depends on the polyphenol structure and the composition of the host microbiota [95].

Beyond SCFA levels, most studies reported an increase in health-promoting bacterial genera. Significant rises in *Faecalibacterium* [65,66], *Akkermansia* [57,59,60,64], *Bifidobacterium* [56,59,60], and *Lactobacillus* [56,65] were observed across multiple trials. Nevertheless, these effects were not uniform. While *Bifidobacterium* abundance increased following juçara [60] and pomegranate juice [59], González-Sarrías et al. [58] observed a reduction after pomegranate extract administration. Similar inconsistency was observed for *Akkermansia muciniphila*, with some studies showing increases [57,59,60,64], while others, such Most et al. [51] and the meta-analysis by Mao et al. [18], did not confirm such effects. These differences highlight the role of host metabolic status and polyphenol matrix in shaping microbial responses.

Regarding microbial diversity, the results were mixed. Most studies, including van der Merwe et al. [67], reported no significant changes in alpha- or beta-diversity despite microbial compositional shifts. However, studies such as Lúcio et al. [63] and Santamarina et al. [65] observed alterations in bacterial community structure, suggesting that polyphenols might influence microbial composition more than richness per se.

An interesting contribution to this discussion comes from studies not included in this systematic review but that are conceptually relevant. Reverri et al. [96] classified participants based on their microbial capacity to metabolize daidzein into ODMA and/or equol. Those capable of producing both metabolites showed a more favorable metabolic profile. Nonetheless, this did not always translate into lower inflammation. In fact, Nakatsu et al. [96] observed elevated levels of IL-6 and hs-CRP in equol producers with insulin resistance. These findings suggest that the clinical impact of polyphenol-derived metabolites depends not only on microbial phenotype but also on the host’s metabolic and immune status.

Altogether, the findings from this systematic review suggest that polyphenol supplementation can beneficially modulate the gut microbiota and increase SCFAs production, particularly butyrate and acetate. However, the direction and magnitude of these effects depend on multiple factors, including the type and matrix of the polyphenol, intervention duration, host metabolic state, and baseline microbiota composition. When compared with data from other systematic reviews and non-included trials, the present findings reinforce the importance of accounting for inter-individual variability in future clinical applications and mechanistic studies.

### 4.4. Effects of Polyphenol Supplementation on Body Weight and BMI

Regarding anthropometric outcomes, the pooled analysis showed no statistically significant effect of polyphenol supplementation on either body weight (SMD = 0.13; 95% CI: −0.43 to 0.69) or BMI (SMD = −0.04; 95% CI: −0.80 to 0.73). These findings suggest that polyphenol intake, in the absence of energy restriction or other lifestyle interventions, is unlikely to elicit meaningful short-term changes in body composition in overweight or obese individuals.

This lack of effect may be partially explained by the diversity of polyphenol sources used across the included trials. For instance, Hou et al. [59] and Fava et al. [56] employed pomegranate juice and wheat aleurone, respectively—both rich in polyphenols such as punicalagins or ferulic acid derivatives—but did not observe significant anthropometric improvements. Similarly, González-Sarrías et al. [58], using a pomegranate extract, reported beneficial changes in oxidative and inflammatory markers, yet no differences in body weight or BMI were noted. Moreover, this lack of effect is likely due to the fact that most included studies did not combine polyphenol supplementation with caloric restriction, structured exercise, or broader lifestyle interventions, strategies known to be essential for achieving meaningful anthropometric changes. Consequently, polyphenols alone, even with promising mechanistic properties, are unlikely to induce clinically relevant reductions in body weight or BMI without concurrent behavioral changes.

In contrast, studies such as Machado et al. [64] and Lúcio et al. [63] combined polyphenol-rich foods like yacon flour or sorghum with structured calorie-restricted diets and indeed reported reductions in weight and fat mass. However, the hypocaloric context of these interventions limits the ability to attribute such effects exclusively to the polyphenol content. The same applies to Santamarina et al. [65], where silymarin, a flavonolignan derived from *Silybum marianum*, was incorporated into a multi-ingredient nutraceutical, leading to reductions in weight and inflammatory markers; but, again, the effect cannot be isolated.

Interestingly, the only study that reported a reduction in adiposity independently of calorie restriction was that by Lee et al. [62], using *Ecklonia cava* polyphenols, which are known to modulate adipogenesis, increase AMPK activation, and improve the Firmicutes/Bacteroidetes ratio. However, this was a secondary analysis with a small sample size, limiting the strength of its conclusions.

From a mechanistic perspective, several polyphenols have demonstrated anti-obesogenic properties in preclinical and clinical studies. Curcumin, for example, exerts anti-adipogenic and thermogenic effects via AMPK activation, the inhibition of lipogenesis, and the upregulation of adiponectin, which together support improved insulin sensitivity and reduced fat accumulation [97,98,99,100]. EGCG, the major catechin in green tea, promotes lipid oxidation, thermogenesis, and appetite suppression, partly by modulating ghrelin and leptin signaling [101,102,103,104]. Resveratrol, found in grapes and red wine, activates SIRT1 and enhances mitochondrial function, thereby improving energy expenditure and insulin sensitivity [105,106,107]. Similarly, quercetin regulates lipid metabolism genes, reduces inflammation, and improves hormonal balance [108,109]. Gallic acid, abundant in berries and wine, inhibits adipocyte differentiation and lipogenesis while stimulating fatty acid oxidation [110,111].

### 4.5. Limitations and Future Directions

Nevertheless, considerable heterogeneity was observed across studies, reflected in wide confidence intervals and high *I*^2^ values. This likely stems from variation in polyphenol source (e.g., pomegranate extract, cranberry, genistein), dosage, formulation (capsules, juices, extracts), duration (ranging from 2 to 24 weeks), and participant characteristics (e.g., BMI, age, comorbidities). Such variability limits the ability to perform detailed dose–response analyses and may dilute pooled effect sizes.

Further limitations include the relatively small sample sizes in several trials, short intervention periods, lack of blinding in some studies, and heterogeneous outcome reporting, particularly regarding antioxidant enzymes and microbiota composition. Specifically, data on gut microbiota composition were highly inconsistent across studies: some reported relative abundance of selected taxa and others presented global diversity indices or only qualitative trends. This variability precluded the possibility of performing a quantitative meta-analysis of microbiota changes. Additionally, the absence of standardized polyphenol quantification and poor reporting of bioavailability data hinders mechanistic interpretations. Moreover, some interventions were relatively short in duration (e.g., 2–3 weeks), which may limit the detection of clinically meaningful effects. While early responses in oxidative or microbial markers were observed in some trials, longer intervention periods are likely required to evaluate sustained changes.

Future research should prioritize well-powered randomized controlled trials with standardized interventions, longer durations, and uniform outcome measurement. Studies should incorporate microbial sequencing techniques and targeted metabolomics to better characterize host–microbiota interactions. Furthermore, stratified analyses by metabolic phenotype and sex could elucidate potential effect modifiers.

Clinically, our findings suggest that polyphenol-rich supplementation may serve as a supportive strategy to mitigate metabolic disturbances in being overweight or obese. While not a replacement for established lifestyle or pharmacological therapies, these bioactive compounds may enhance gut health, reduce oxidative damage, and counteract low-grade inflammation, thereby contributing to improved metabolic resilience.

## 5. Conclusions

Polyphenol-rich interventions appear to offer beneficial effects on gut-derived inflammation and oxidative stress in individuals who are overweight or obese, notably through reductions in LPS and increases in SCFA production and improvements in antioxidant enzyme activity. However, the effects on pro-inflammatory cytokines such as IL-6 and TNF-α were inconsistent and not statistically significant in the meta-analysis, highlighting the current lack of robust evidence for systemic anti-inflammatory effects. Similarly, no conclusive impact was observed on body weight or BMI, likely due to heterogeneity in study designs, intervention durations, and lack of concurrent lifestyle modifications. Overall, these findings support the potential adjunctive role of polyphenols in metabolic health, while underscoring the need for well-designed, mechanistically focused trials.

## Figures and Tables

**Table 1:** Study and Participant Characteristics.

Author (Year)	Country	Study Design	Duration	*n*	Age	Sex (M/F)	BMI (kg/m^2^)
Fava et al. (2022) [56]	Italy	RCT, double-blind, placebo-controlled	4 weeks	67	47.2	31 M/36 F	31.4
González-Sarrías et al. (2017) [58]	Spain	RCT, double-blind, placebo-controlled, crossover	6 months (two 3-week phases + washout)	49	46,2	32 M/17 F	30.4
Guevara-Cruz et al. (2020) [57]	Mexico	RCT, double-blind, placebo-controlled, parallel	8 weeks	45	44	NR	34.1
Hou et al. (2024) [59]	China	RCT, double-blind, placebo-controlled	3 weeks	67	47	35 M/32 F	28
Jamar et al. (2020) [60]	Brazil	RCT, double-blind, placebo-controlled	6 weeks	34	46.5	14 M/21 F	34.4
Jin et al. (2024) [61]	China	RCT, double-blind, placebo-controlled	24 weeks	83	42.9	49 M/34 F	27.3
Lee et al. (2022) [62]	South Korea	RCT (secondary analysis)	12 weeks	40	37.4	17 M/23 F	27.7
Lúcio et al. (2023) [63]	Brazil	RCT, single-blind, controlled	8 weeks	21	25.6 ± 4.6 years	21M	28.5
Machado et al. (2020) [64]	Brazil	RCT, double-blind, placebo-controlled	6 weeks	26	31.3	11 M/15 F	30.4
Santamarina et al. (2025) [65]	Brazil	RCT, double-blind	90 days	77	54.5	21 M/56 F	27.8
Solch-Ottaiano et al. (2022) [66]	USA	RCT, double-blind, placebo-controlled, crossover	2 weeks per intervention (12 weeks total with washout)	36	35.4	10 M/26 F	37.4
van der Merwe et al. (2021) [67]	USA	RCT, double-blind, placebo-controlled	16 weeks (+4 weeks in subgroup)	57	36.2	57 F	30.6
Vitaglione et al. (2015) [68]	Italy	RCT, placebo-controlled, parallel	8 weeks	68	38.5 years	11 M/25 F	29.8

BMI, Body Mass Index; F, Female; M, Male; RCT, Randomized Clinical Trial.

**Table 2:** Interventions, Outcomes, and Main Conclusions.

Author (Year)	Intervention		Comparator	Study Focus	Methods of Analysis	Main Conclusions
	Polyphenol Type	Polyphenol Dose				
Fava et al. (2022) [56]	Aleurone (ferulic, cinnamic, benzoic acids)	27 g/day aleurone	Placebo (cellulose)	CVD biomarkers, gut microbiota, metabolites in overweight/obese adults	qPCR, FCM-FISH, 16S rRNA, ELISA, untargeted metabolomics	↑ *Bifidobacterium*, *Lactobacillus*; no change in inflammatory/oxidative markers
González-Sarrías et al. (2017) [58]	Pomegranate polyphenols (extract): Punicalin, valoneic acid dilactone, sanguisorbic acid, gallagic acid dilactone, ellagic acid, gallic acid	160 (1 capsule) or 640 mg (4 capsules) phenolics/day	Placebo (maltodextrin)	Gut microbiota, urolithin metabotypes, inflammation, oxidative stress	qPCR, 16S rRNA, HPLC-DAD, ELISA	↓ LDLc/oxLDL in UM-B individuals; *Gordonibacter* correlated with urolithin production
Guevara-Cruz et al. (2020) [57]	Genistein (isoflavone)	50 mg/day	Placebo	Insulin sensitivity, gut microbiota, metabolic endotoxemia in obesity	16S rRNA, blood glucose/lipids, HOMA-IR, muscle AMPK phosphorylation, serum metabolomics	↑ *Akkermansia*, insulin sensitivity; ↓ LPS; AMPK activation in muscle
Hou et al. (2024) [59]	Pomegranate polyphenols (juice)	200 mL/day juice	Placebo (flavored drink)	Gut microbiota/metabolites in overweight/obese individuals	16S rRNA, HPLC (polyphenols), GC–MS (SCFAs)	↑ *Akkermansia*, *Bifidobacterium*, SCFAs, urolithins; no anthropometric changes
Jamar et al. (2020) [60]	Anthocyanins (cyanidin-3-glucoside, rutinoside)	5 g/day lyophilized juçara	Placebo (maltodextrin)	Prebiotic potential of juçara berry on gut microbiota and SCFAs in obese individuals	qPCR (Akkermansia, Bifidobacterium), GC-FID (SCFAs), serum LPS	↑ *Akkermansia*, *Bifidobacterium*, acetate; no LPS changes
Jin et al. (2024) [61]	Silymarin (flavonolignans)	103.2 mg/day (4 tablets/day)	Placebo (dextrin)	Effects of silymarin on liver stiffness and gut microbiota in MASLD patients	FibroScan for liver stiffness/steatosis, 16S rRNA sequencing for gut microbiota, blood biochemical tests	↓ Liver stiffness, GGT; ↑ *Oscillospiraceae*; no hepatic steatosis improvement
Lee et al. (2022) [62]	Phlorotannins from *Ecklonia cava*	360 mg/day	Placebo	EP effects on adiposity and gut microbiota in abdominal obesity	16S rRNA, anthropometrics, oxidative stress markers, Tax4Fun	↓ Adiposity, oxidative stress; ↑ *Butyricimonas*, *Gordonibacter*; improved Firmicutes/Bacteroidetes ratio
Lúcio et al. (2023) [63]	Proanthocyanidins, 3-deoxyanthocyanidins	40 g/day SC319 sorghum	Whole wheat (38 g/day) + diet (−500 kcal/day)	Effects on gut microbiota, anthropometric markers, and inflammatory markers in overweight men	DXA, ELISA (IL-6/IL-10/TNF-α), 16S rRNA, qPCR, HPLC (SCFAs), fecal pH	↓ Weight, body fat; modulated microbiota (↓ *Clostridium*); ↑ IL-6 in wheat group
Machado et al. (2020) [64]	Chlorogenic acid	25 g/day yacon flour	Placebo (control drink) + diet (−500 kcal/day)	Effects on intestinal permeability, fecal SCFAs, oxidative stress, and inflammation in overweight/obese adults	HPLC (SCFAs, lactulose/mannitol), FRAP, carbonyls, catalase, GST, MDA, NO, CRP, leukocytes, NLR, PLR	↑ Plasma antioxidants, *Akkermansia*; ↓ carbonyls; fecal SCFAs ↓ (weight loss effect)
Santamarina et al. (2025) [65]	Silymarin	4 capsules/day	Nutraceutical blend (FOS + GOS + β-glucans + minerals)	Gut microbiota, inflammation, sleep in overweight adults	16S rRNA (QIIME 2), CBA (cytokines), HPLC (silymarin), PSQI/ESS/MSQ-BR/BRUMS	↑ *Faecalibacterium*, *Lactobacillus*; ↓ weight, TNF-α/IL-10; improved sleep, Silymarin enhanced anti-inflammatory effects
Solch-Ottaiano et al. (2022) [66]	Proanthocyanidins, anthocyanins, phenolics	480 mL/day; 11 mg anthocyanins, 407 mg phenolics, 535 mg proanthocyanidins	Placebo (matched drink, no polyphenols)	Gut permeability, microbiota, and inflammation after aspirin challenge in obese adults	LC-MS/MS (sugar probes), 16S rRNA (QIIME 2), qPCR, ELISA (hs-CRP/IL-6/TNF-α/zonulin)	↑ *Faecalibacterium prausnitzii*, *Eggerthella lenta*; no gut permeability/inflammation changes
van der Merwe et al. (2021) [67]	Quercetin, kaempferol, catechin, epicatechin, chlorogenic acid, rutin, hesperidin, narirutin	6 capsules/day JuicePlus+	Placebo (cellulose); habitual breakfast	Gut microbiota, SCFAs, glucose metabolism, inflammation, permeability	16S rRNA (QIIME), ion chromatography (SCFAs), Luminex (cytokines), OGTT, DXA	↓ *Bacteroides*; ↑ butyrate; no effect on α/β-diversity, lipids or inflammation; improved glucose clearance with FVC
Vitaglione et al. (2015) [68]	Ferulic acid, sinapic acid, caffeic acid, p-coumaric acid	70 g/day whole grain wheat	Refined wheat products	Polyphenol bioavailability, gut microbiota, inflammation in overweight/obese	HPLC-MS/MS (phenolic acids), Luminex (cytokines), 16S rRNA (MiSeq), bioelectrical impedance	↑ Ferulic acid metabolites; ↓ TNF-α, ↑ IL-10; ↑ Bacteroidetes/Firmicutes; no weight/lipid changes

AMPK, AMP-activated protein kinase; BRUMS, Brunel Mood Scale; CBA, Cytometric Bead Array; CVD, Cardiovascular Disease; DAD, Diode Array Detector; DXA, Dual-energy X-ray Absorptiometry; ELISA, Enzyme-Linked Immunosorbent Assay; EP, Ecklonia cava Polyphenol; FCM-FISH, Flow Cytometry-Fluorescence In Situ Hybridization; FOS, Fructooligosaccharides; FRAP, Ferric Reducing Ability of Plasma; GC-FID, Gas Chromatography-Flame Ionization Detection; GC–MS, Gas Chromatography-Mass Spectrometry; GOS, Galactooligosaccharides; GST, Glutathione S-Transferase; HOMA-IR, Homeostatic Model Assessment for Insulin Resistance; HPLC, High-Performance Liquid Chromatography; HPLC-DAD, High-Performance Liquid Chromatography-Diode Array Detector; hs-CRP, High-Sensitivity C-Reactive Protein; IL-6, Interleukin-6; IL-10, Interleukin-10; LC-MS/MS, Liquid Chromatography-Tandem Mass Spectrometry; LDLc, Low-Density Lipoprotein Cholesterol; LPS, Lipopolysaccharide; MASLD, Metabolic Dysfunction-Associated Steatotic Liver Disease; MDA, Malondialdehyde; NLR, Neutrophil-to-Lymphocyte Ratio; NO, Nitric Oxide; OGTT, Oral Glucose Tolerance Test; oxLDL, Oxidized Low-Density Lipoprotein; PLR, Platelet-to-Lymphocyte Ratio; PSQI, Pittsburgh Sleep Quality Index; qPCR, Quantitative Polymerase Chain Reaction; QIIME 2, Quantitative Insights Into Microbial Ecology 2; SCFAs, Short-Chain Fatty Acids; TNF-α, Tumor Necrosis Factor-alpha; UM-B, Urolithin Metabotype B. The use of ↑ and ↓ in the text refers to increases and decreases, respectively.

## Data Availability

Data are contained within the article.

## Supplementary Materials

The following supporting information can be downloaded at https://www.mdpi.com/article/10.3390/nu17152468/s1: Table S1: Search strategy, Table S2: PRISMA Checklist. Figure S1: Funnel plots for inflammatory biomarkers (LPS, CRP, IL-6, and TNF-α), Figure S2: Sensitivity analysis for inflammatory biomarkers, Figure S3: Funnel plots for oxidative stress and antioxidant biomarkers (MDA, oxLDL, SOD, and CAT), Figure S4: Sensitivity analysis for oxidative stress and antioxidant biomarkers, Figure S5: Funnel plots for short-chain fatty acids (acetate, propionate, and butyrate), Figure S6: Sensitivity analysis for short-chain fatty acids, Figure S7: Funnel plots for body weight and BMI, Figure S8: Sensitivity analysis for body weight and BMI.

## Abbreviations

The following abbreviations are used in this manuscript:

BMI	Body Mass Index
CAT	Catalase
CRP	C-reactive Protein
GC–MS	Gas Chromatography–Mass Spectrometry
GPx	Glutathione Peroxidase
HPLC	High-Performance Liquid Chromatography
IL-6	Interleukin 6
ITT	Intention-to-Treat
LPS	Lipopolysaccharide
MeSH	Medical Subject Headings
MDA	Malondialdehyde
PP	Per-protocol
qPCR	Quantitative Polymerase Chain Reaction
RCTs	Randomized Controlled Trials
SCFA	Short-Chain Fatty Acids
SCFAs	Short-Chain Fatty Acids
SOD	Soperoxide Dismutase
TNF-α	Tumor Necrosis Factor Alpha
