# Supplementary material for: Effect of Polyphenol-Rich Interventions on Gut Microbiota and Inflammatory or Oxidative Stress Markers in Adults Who Are Overweight or Obese: A Systematic Review and Meta-Analysis
Source: Nutrients. 2025 Jul 29;17(15):2468. doi: 10.3390/nu17152468 (PMC12348198; doi:10.3390/nu17152468)
Supplement: Supplementary file 1 [file nutrients-17-02468-s001.zip › Table S1 - Search strategy.pdf]

Table S1. Search strategy.

Databases searched until 16th July 2025.
Strategy for Web of Science: TS=(polyphenol* OR flavonoid* OR "phenolic compound*" OR "phenolic acid*" OR resveratrol OR curcumin OR quercetin OR catechin* OR anthocyanin* OR stilbene* OR lignan* OR tannin* OR EGCG OR genistein OR hesperidin) AND TS=("gut microbiota" OR "intestinal flora" OR dysbiosis OR SCFA OR "short-chain fatty acid*" OR Lactobacillus OR Bifidobacteria OR Bacteroides) AND TS=(inflammation OR cytokine* OR CRP OR IL-6 OR TNF OR ROS OR MDA OR SOD OR CAT OR LPS OR "oxidized LDL" OR antioxidant* OR endotoxemia) AND TS=(obes* OR overweight OR "body fat" OR "waist circumference" OR BMI OR "metabolic syndrome") AND TS=("randomized controlled trial" OR randomized OR RCT OR "clinical trial" OR "intervention study" OR "placebo-controlled") (All Fields) (No limitations were used).
**Results: 128**
Strategy for PubMed: ("Polyphenols"[Mesh] OR polyphenols OR flavonoids OR "Phenolic compounds" OR "Phenolic acids" OR "Anthocyanins" OR "Resveratrol" OR "Curcumin" OR "Quercetin" OR "EGCG" OR "Epigallocatechin gallate" OR "Chlorogenic acid" OR "Tannins" OR "Catechins" OR "Stilbenes" OR "Lignans" OR "Proanthocyanidins" OR "Curcuminoids" OR "Hesperidin" OR "Naringenin" OR "Myricetin" OR "Kaempferol" OR "Fisetin" OR "Baicalein" OR "Baicalin" OR "Apigenin" OR "Luteolin" OR "Genistein" OR "Daidzein" OR "Silymarin" OR "Gallocatechin" OR "Theaflavins" OR "Thearubigins" OR "Hydroxytyrosol" OR "Oleuropein" OR "Gallic acid" OR "Ferulic acid" OR "Caffeic acid" OR "Vanillic acid" OR "Piceatannol" OR "Secoisolariciresinol" OR "Enterolactone" OR "Enterodiol") AND ("Gastrointestinal Microbiome"[Mesh] OR "gut microbiota" OR "gut microbiome" OR "intestinal flora" OR "fecal microbiota" OR "microbial diversity" OR "bacterial composition" OR "dysbiosis" OR "microbiota-derived metabolites" OR "short-chain fatty acids" OR SCFA OR "Lactobacillus" OR "Bifidobacteria" OR "Faecalibacterium prausnitzii" OR "Akkermansia muciniphila" OR “Bacteroides”) AND ("Inflammation"[Mesh] OR "Oxidative Stress"[Mesh] OR cytokine* OR CRP OR IL-6 OR TNF OR ROS OR MDA OR SOD OR CAT OR LPS OR "oxidized LDL" OR antioxidant* OR endotoxemia) AND ("Obesity"[Mesh] OR obes* OR overweight OR "body fat" OR "waist circumference" OR BMI OR "metabolic syndrome") AND ("Randomized Controlled Trial"[pt] OR randomized OR RCT OR "clinical trial" OR "intervention study") (No limitations were used).
**Results: 42**
Strategy for SCOPUS: TITLE-ABS-KEY(polyphenol* OR flavonoid* OR "phenolic compound*" OR "phenolic acid*" OR resveratrol OR curcumin OR quercetin OR catechin* OR anthocyanin* OR stilbene* OR lignan* OR tannin* OR EGCG OR genistein OR hesperidin) AND TITLE-ABS-KEY("gut microbiota" OR "intestinal flora" OR dysbiosis OR SCFA OR "short-chain fatty acid*" OR Lactobacillus OR Bifidobacteria OR Bacteroides) AND TITLE-ABS-KEY(inflammation OR cytokine* OR CRP OR IL-6 OR TNF OR ROS OR MDA OR SOD OR CAT OR LPS OR "oxidized LDL" OR antioxidant* OR endotoxemia) AND TITLE-ABS-KEY(obes* OR overweight OR "body fat" OR "waist circumference" OR BMI OR "metabolic syndrome") AND TITLE-ABS-KEY("randomized controlled trial" OR randomized OR RCT OR "clinical trial" OR "intervention study" OR "placebo-controlled")
**Results: 141**
Strategy for Cochrane: ((polyphenol* OR flavonoid* OR "phenolic compound*" OR "phenolic acid*" OR anthocyanin* OR resveratrol OR curcumin OR quercetin OR catechin* OR EGCG OR tannin* OR lignan* OR genistein OR hesperidin OR silymarin OR "gallic acid" OR "ferulic acid" OR "caffeic acid") AND ("gut microbiota" OR "intestinal microbiota" OR "gut microbiome" OR dysbiosis OR "bacterial diversity" OR "short-chain fatty acid*" OR SCFA OR Lactobacillus OR Bifidobacterium OR Faecalibacterium OR Akkermansia OR Bacteroides) AND (inflammation OR "oxidative stress" OR cytokine* OR CRP OR IL-6 OR TNF OR ROS OR MDA OR SOD OR CAT OR LPS OR "oxidized LDL" OR antioxidant* OR endotoxemia) AND (obes* OR overweight OR "body fat" OR BMI OR "metabolic

syndrome") AND (randomized OR "randomised controlled trial" OR RCT OR "clinical trial")):ti,ab,kw
**Results: 68**
