# Supplementary figures and images for: Effect of Polyphenol-Rich Interventions on Gut Microbiota and Inflammatory or Oxidative Stress Markers in Adults Who Are Overweight or Obese: A Systematic Review and Meta-Analysis
Source: Nutrients. 2025 Jul 29;17(15):2468. doi: 10.3390/nu17152468 (PMC12348198; doi:10.3390/nu17152468)

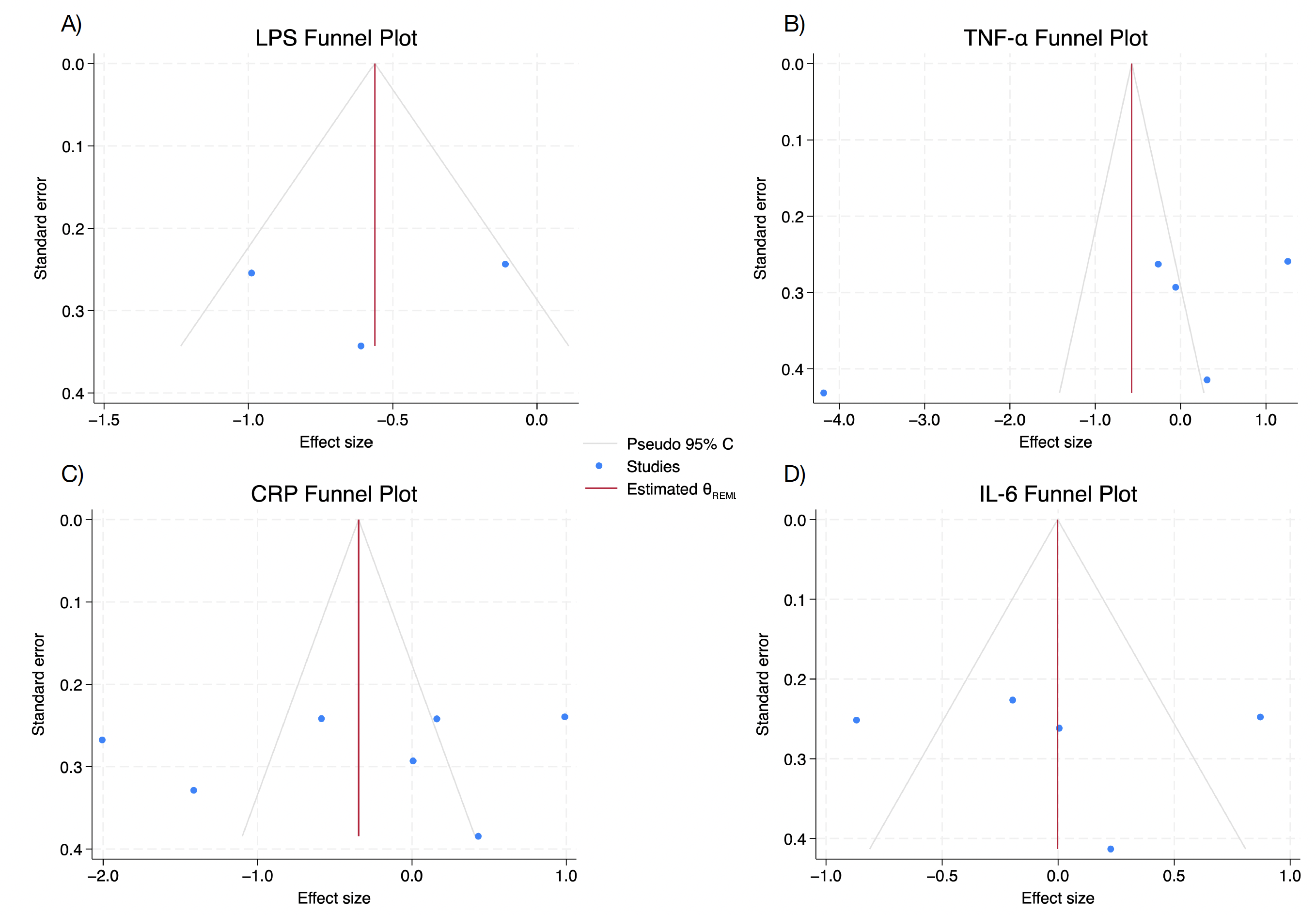

Supplement: Supplementary file 1 [file nutrients-17-02468-s001.zip › Figure S1.png]

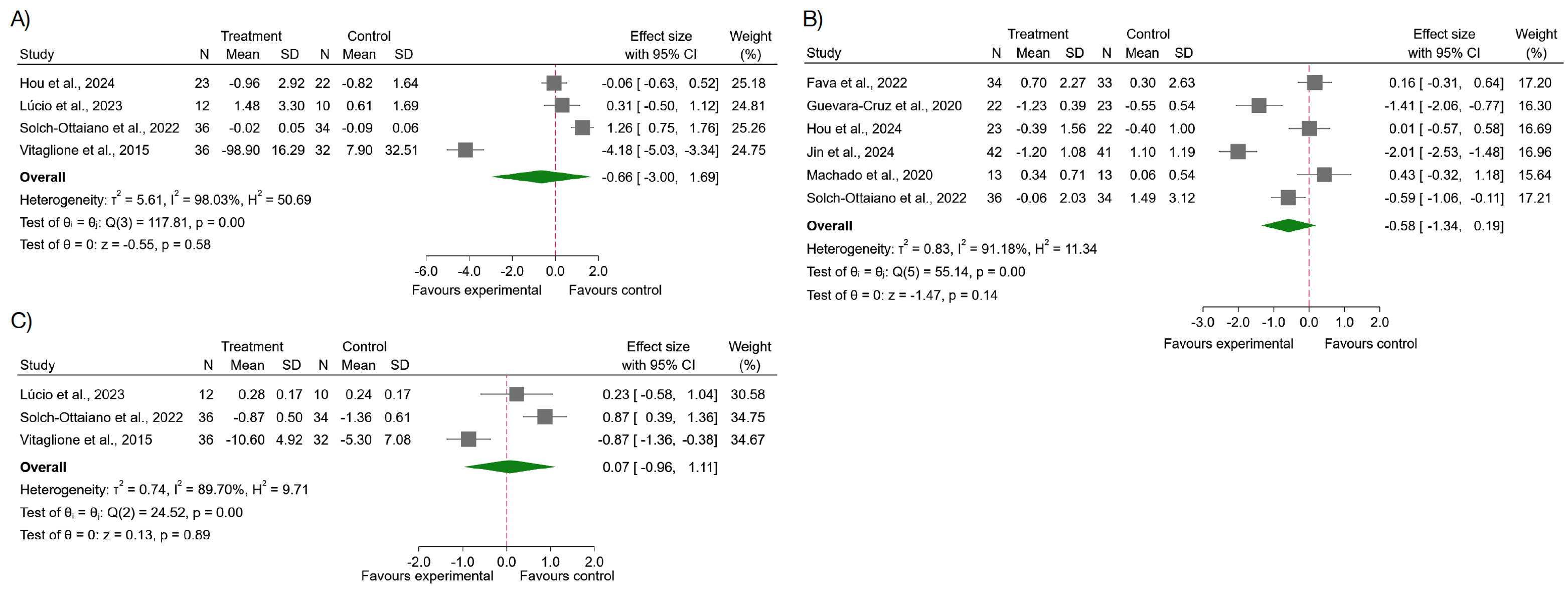

Supplement: Supplementary file 1 [file nutrients-17-02468-s001.zip › Figure S2.png]

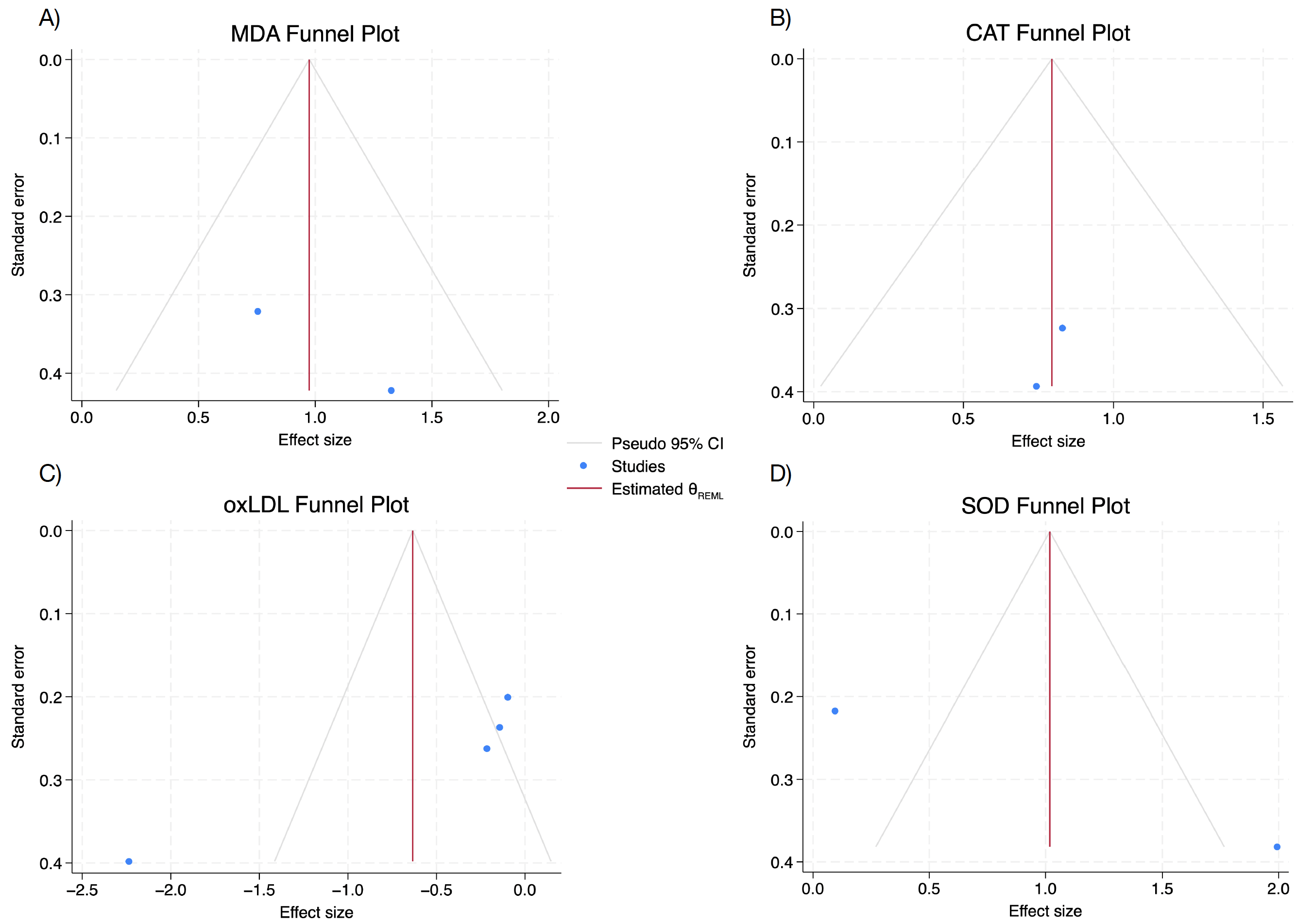

Supplement: Supplementary file 1 [file nutrients-17-02468-s001.zip › Figure S3.png]

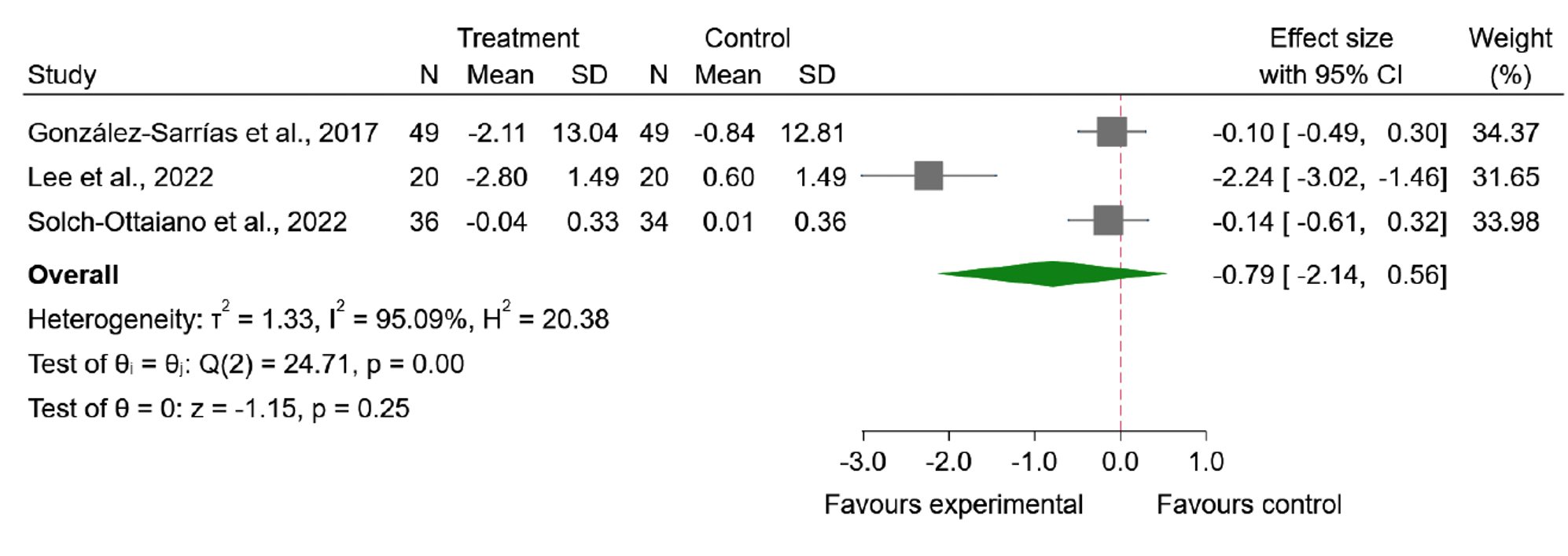

Supplement: Supplementary file 1 [file nutrients-17-02468-s001.zip › Figure S4.png]

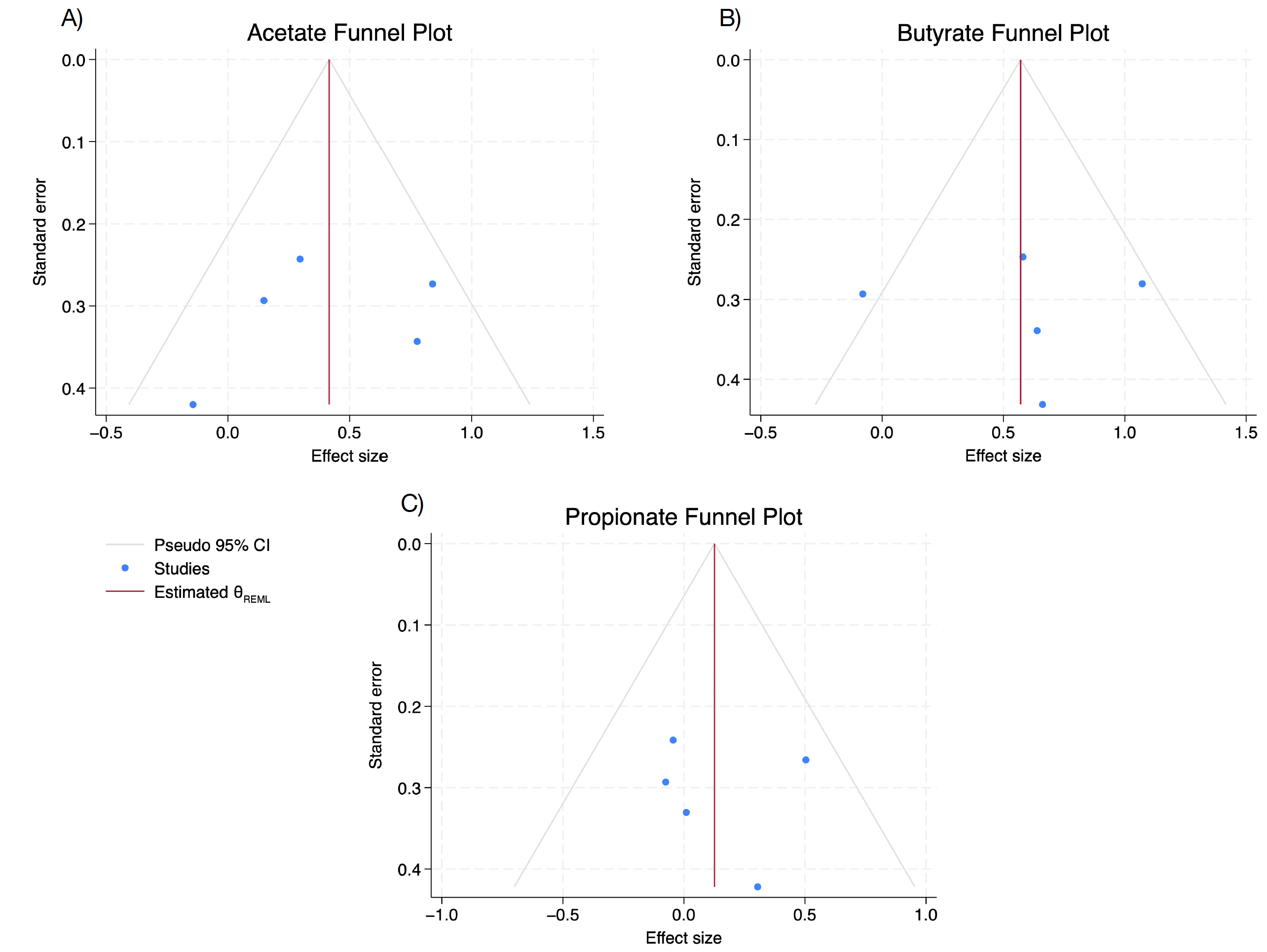

Supplement: Supplementary file 1 [file nutrients-17-02468-s001.zip › Figure S5.png]

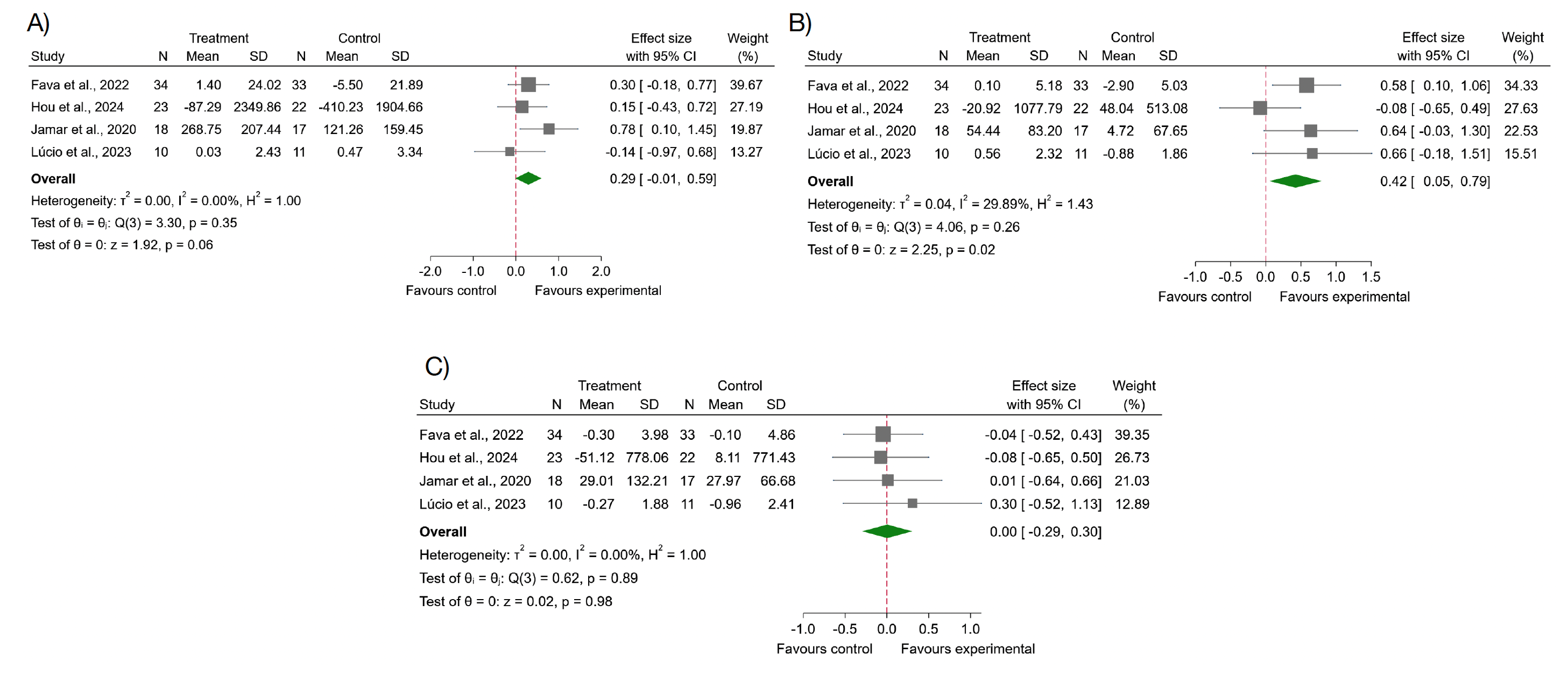

Supplement: Supplementary file 1 [file nutrients-17-02468-s001.zip › Figure S6.png]

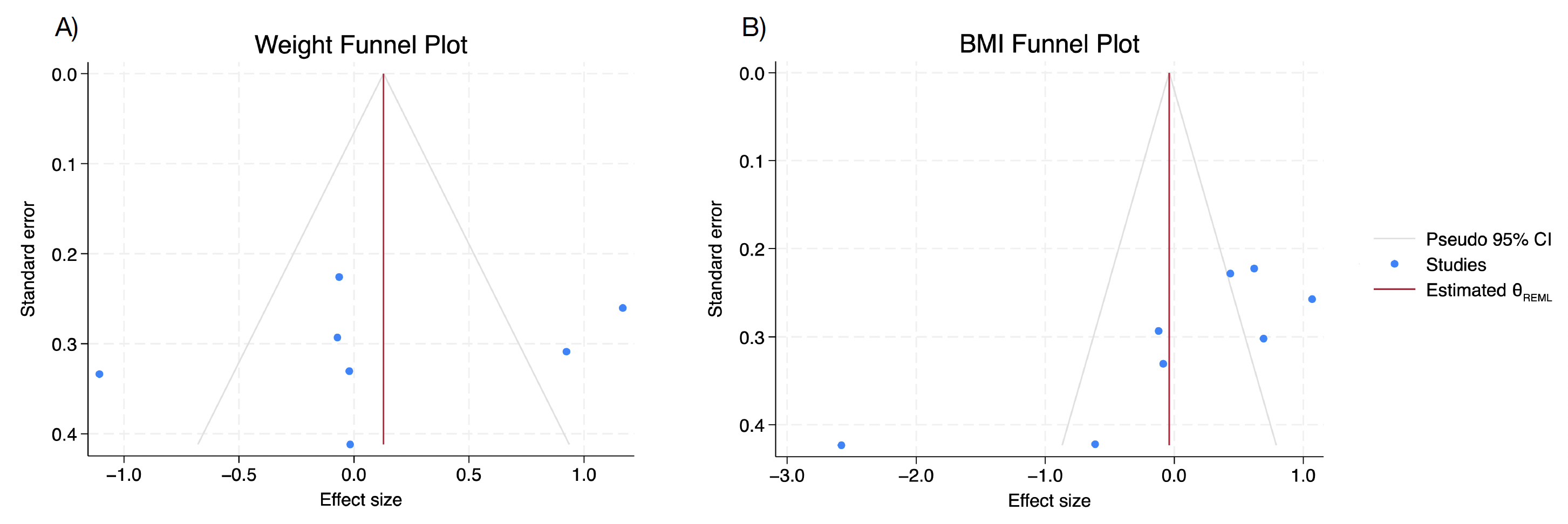

Supplement: Supplementary file 1 [file nutrients-17-02468-s001.zip › Figure S7.png]

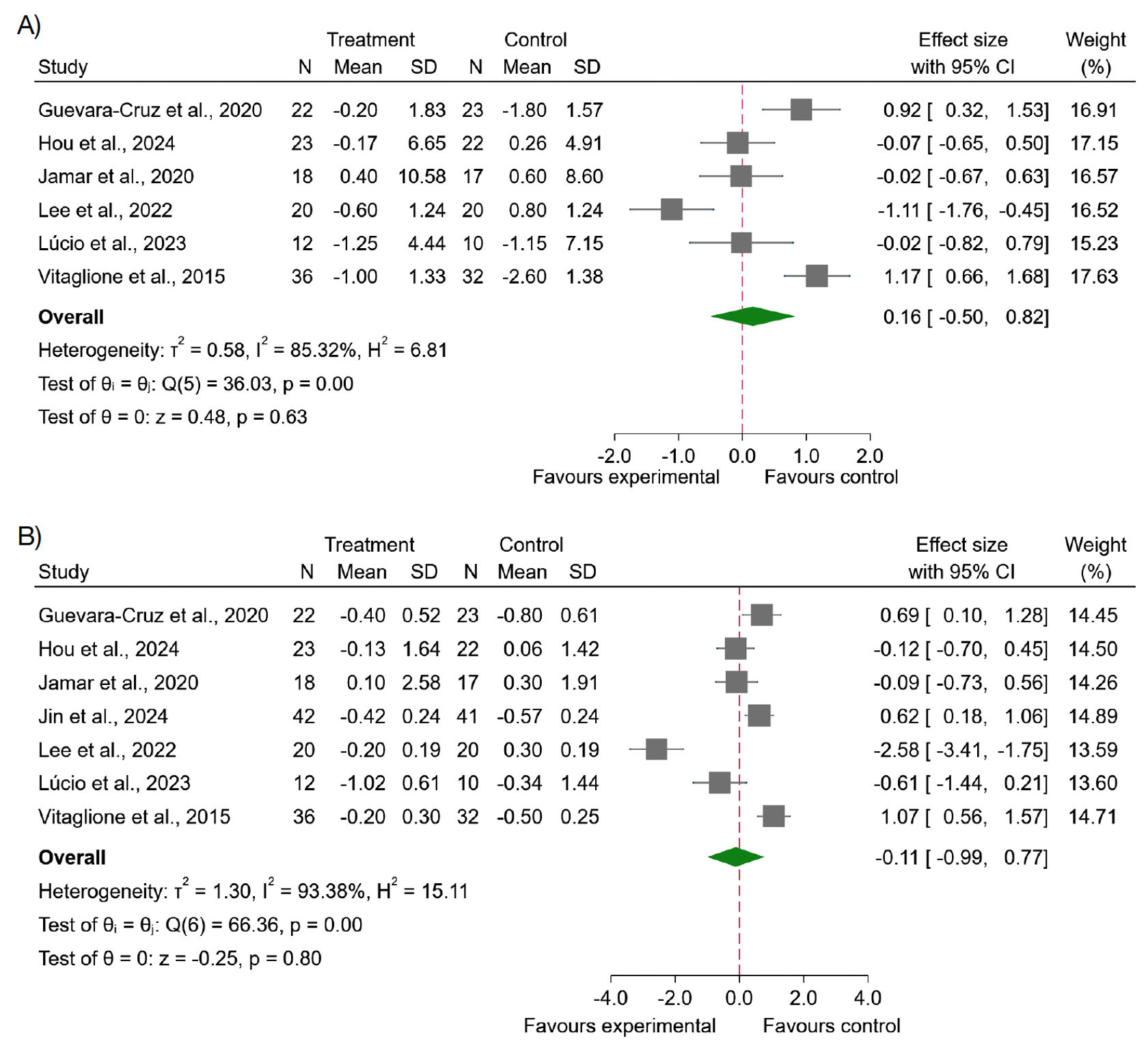

Supplement: Supplementary file 1 [file nutrients-17-02468-s001.zip › Figure S8.png]
